# Developing Quick Screening Method to Identify Rice Cultivars with Unique Aromatic Features

**DOI:** 10.1021/acsomega.6c02582

**Published:** 2026-06-01

**Authors:** Heena Rani, Rahul Sen, Christian De Guzman, Scott Lafontaine

**Affiliations:** † Department of Food Science, 3341 University of Arkansas, 2650 N Young Ave, Fayetteville, Arkansas 72704, United States; ‡ Division of Agriculture, Rice Research and Extension Center, University of Arkansas, Stuttgart, Arkansas 72160, United States

## Abstract

Aroma is a primary determinant of rice quality and market value, yet its evaluation in breeding programs remains constrained by labor-intensive milling, cooked-grain sensory methods, binary screening assays, and the limited seed availability of early generation selection. Moreover, aromatic rice breeding has historically focused narrowly on 2-acetyl-1-pyrroline-mediated popcorn aroma, potentially overlooking valuable alternative aromatic profiles. In this study, we developed a rapid sensory phenotyping approach for paddy rice that enables quantitative assessment of the aroma intensity and qualitative aroma characterization without milling or cooking. A diverse panel of 126 rice genotypes was evaluated using 1 g of ground paddy rice heated under controlled conditions coupled with sensory analysis and targeted HS-SPME-GC-MS/MS quantification of 164 volatile compounds. The method discriminated the aroma intensity and enabled characterization of aroma quality. Hierarchical clustering integrating sensory and chemical data resolved five distinct aroma classes, including popcorn-dominant, fruity-floral, nutty-grainy, woody-floral, and oxidation-driven phenotypes. While 2AP showed the strongest association with popcorn aroma and overall intensity (*r* = 0.50), several high-intensity genotypes exhibited minimal 2AP, yet strong aroma perception driven by esters, alcohols, indole, and ketones. Interestingly, two genotypes (R125 and R126) showed strong popcorn perception despite much lower 2AP than typical aromatic rice, indicating the contribution of non-2AP popcorn-like aroma drivers. Conversely, genotypes with elevated lipid oxidation aldehydes exhibited high volatile abundance but poor aroma quality characterized by rancid, phenolic, and musty notes. These results demonstrate that superior rice aroma is a multivariate trait and is not related to only 2AP. The rapid phenotyping framework presented here provides breeding programs with an employable, information-rich tool for early generation screening, accelerating the identification of aromatic rice cultivars with expanded sensory diversity.

## Graphical abstract

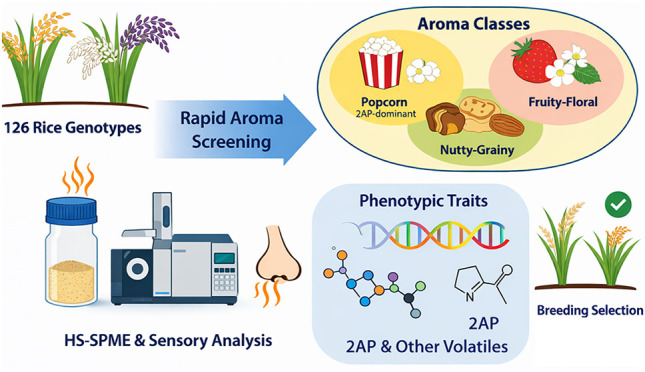

## 1 Introduction

Rice (*Oryza sativa* L.) is one of the most important crops worldwide, serving as the primary food for over half of the global population.^1^ In 2025, world rice production reached a record high of approximately 551 million tonnes, reflecting rice’s critical role in global food security (FAO 2025). Asia remains the largest producing and consuming region, but rice is also significant in the United States, both as a food and an agricultural commodity. The U.S. produces a smaller share of the world’s rice (roughly 2%) yet consistently ranks among the top exporters (USDA ERS 2025). Within the U.S., rice is economically vital in several states especially Arkansas, which is the nation’s leading rice producer. Arkansas alone accounted for about 49% of U.S. rice production in 2023, underlining the crop’s importance to both the regional economy and national food supply.^2^

Beyond agronomic performance, rice quality is defined by several traits, among which aroma has emerged as a dominant driver of consumer perception and preference. A growing body of recent literature reports that the aromatic profile of rice strongly influences consumer choice, market differentiation, and perceived eating quality.^3–5^ Even small differences in aromatic composition can determine consumer acceptance or rejection of a rice cultivar,^3,6^ elevating aroma from a secondary trait to a primary criterion distinguishing rice varieties in both domestic and global markets.

High-quality aromatic varieties, especially Basmati and Jasmine rice, often command price premiums in the marketplace due to their desirable aroma. Basmati rice, prized for its characteristic popcorn-like aroma, can retail at prices nearly twice those of conventional nonaromatic rice, illustrating the strong economic value associated with superior aroma.^7^ However, reliance on traditional aromatic varieties is not universally viable. These cultivars evolved under specific photoperiod, climate, and agronomic conditions and are largely confined to production regions in Asia,^8^ when grown outside their native environments, these cultivars frequently exhibit reduced yield, inconsistent aroma expression, or compromised grain quality.^9^ Consequently, much of the growing U.S. demand for aromatic rice has historically been met through imports. To reduce dependence on imports and cater to local preferences, U.S. breeders have responded by developing locally adapted aromatic cultivars. Early efforts began with the release of “Della” in Louisiana in 1973, followed by improved derivatives such as “Dellrose” that retained aroma while improving agronomic performance.^10^ Subsequent breeding efforts expanded aromatic rice production across the southern U.S., including the release of including jasmine-type releases in Texas (Jasmine 85).^11^ California-developed basmati- and jasmine-type cultivars (e.g., A-201,^12^ Calmati-201,^13^ Calmati-202,^14^ Calaroma-201 ^15^), and a series of jasmine-style cultivars from the LSU AgCenter (“Jazzman,”^16^ “Jazzman-2,”^17^ and “CLJ01”^18^). More recently, Arkansas’s breeding program introduced “ARoma 22” a Jasmine-type aromatic long-grain cultivar adapted to the U.S. Mid-South environment.^19^

Despite these advances, domestically developed aromatic varieties often still fall short of matching the sensory quality and consistency of the best imported rice, limiting their competitiveness in the premium markets. As a result, breeding programs face increasing pressure to continually enhance aroma quality and diversify aromatic profiles. Progress toward this goal is constrained by a fundamental bottleneck: the lack of rapid, less labor-intensive phenotyping tools capable of capturing aroma intensity and quality across breeding populations at the early selection stages. Addressing this gap is essential for accelerating the identification and deployment of next-generation aromatic rice cultivars.

Current methods for evaluating rice aroma, however, present significant challenges in breeding context. A review of rice aroma literature conducted as part of this study shows that the majority of published sensory evaluations or aroma composition analysis rely on milled rice or cooked form using relatively large sample quantities (Table S1), reflecting an emphasis on end-point consumer acceptance rather than early generation screening suitability.^20^ This prevailing focus introduces practical limitations for breeding applications. First, cooked-rice evaluations require prior milling (dehusking and polishing), adding an additional processing step that is often impractical or infeasible when seed quantities are limited as this process reduces measurable weights by 20–30% and standardized milling equipment is needed. Second, standardized cooking protocols demand relatively large grain quantities (Table S1), which are rarely available for early generation lines. Third, cooking-based sensory assessments are time and labor-intensive, limiting throughput. Finally, physiological constraints such as olfactory fatigue,^21^ which reduce even trained panelists’ capacity to reliably evaluate several samples per session.^22^

At early stages of selection, breeders require a rapid yet informative screening tool that enables the identification of promising lines based on aroma presence, relative intensity, and general sensory character. Breeding programs have historically favored simple, low-cost assays that can be deployed across large populations, as exemplified by the long-standing use of the 1.7% KOH test.^23^ However, the binary nature of this assay severely limits its ability to discriminate among genotypes differing in aroma intensity or qualitative profiles. The absence of rapid, small-sample screening approaches that combine high throughput with semiquantitative and descriptive sensory resolution represents a critical methodological gap. To address this gap, we developed a novel aroma phenotyping method that captures both aroma intensity and sensory character using only 1 g of ground paddy rice samples, eliminating the need for milling and significantly reducing sample preparation losses. Furthermore, the absence of cooking steps reduces both the processing time and labor, allowing rapid sample turnaround and increased throughput. The workflow relies on minimal sample preparation (grinding and controlled heating) and does not require specialized milling equipment, making it more accessible and scalable across breeding programs with varying resource availabilities. Collectively, these features enable efficient screening of large germplasm populations while preserving limited seed material, representing a substantial improvement over conventional cooked-rice evaluation methods.

Beyond methodological efficiency, this study also seeks to reframe how superior rice aromas are defined. Aromatic rice breeding has historically emphasized the popcorn-like note associated with 2-acetyl-1-pyrroline (2AP), yet rice aroma is inherently complex and arises from diverse volatile compounds spanning multiple chemical classes that contribute floral, nutty, grainy, woody, and other sensory attributes.^24^ We hypothesize that high-aroma intensity and consumer appeal are not exclusively linked to the classic popcorn profile and that cultivars expressing alternative aromatic signatures may be equally or even more desirable. By integrating rapid sensory screening with descriptor-based evaluation and confirmatory HS-SPME-GC-MS analysis, this study aims to identify rice genotypes in which a strong aroma intensity is driven by distinct aromatic features beyond 2AP alone. These genotypes offer opportunities to expand aromatic diversity in breeding programs and highlight the need to define rice aroma based on integrated sensory and chemical attributes rather than relying on a single marker compound.

## 2 Materials and Methods

### 2.1 Material

A total of 126 rice (*Oryza sativa* L.) genotypes were evaluated in this study. Seed material was obtained primarily from the USDA Germplasm Resources Information Network (GRIN) in Arkansas, USA. The panel represented a highly diverse collection encompassing elite cultivars, breeding lines, mapping population parents, mutants, and landraces originating from multiple geographic regions worldwide, including Asia, Africa, Europe, North America, South America, and Oceania. Countries and regions represented included, but were not limited to, the United States, China, India, Japan, the Philippines (IRRI), Pakistan, Bangladesh, Vietnam, Laos, Malaysia, Indonesia, Taiwan, South Korea, Iran, Iraq, Nepal, Myanmar, Turkey, Italy, Spain, Hungary, Senegal, Ghana, Brazil, Argentina, Peru, Mexico, Panama, Haiti, Mongolia, Australia, and six accessions of uncertain origin. The varieties were grown in Stuttgart, Arkansas, in 2024 and planted in panicle rows with two rows of each genotype.

### 2.2 Grain Morphology and Starch Physicochemical Properties of Rice

Whole kernel grain characteristics, including kernel length, width, length-to-width ratio, and color, were measured on brown rice using a Vibe QM3 Rice Analyzer (Vibe, Capitola, CA, USA). For determination of apparent amylose content and gelatinization properties, paddy rice samples were first dehusked and finely ground.

Amylose content was determined using an iodine-binding assay based on the method of Juliano,^25^ adapted for high-throughput analysis in a 96-well microplate format. Approximately 15 mg of rice flour was placed into 2 mL safe-lock tubes and dispersed in 150 μL of 95% ethanol, followed by the addition of 1.3 mL of 1 N sodium hydroxide. Samples were incubated at 95 °C for 20 min in a thermomixer (Thermomixer C, Eppendorf, Enfield, CT, USA) with continuous agitation (850 rpm) to ensure complete solubilization of starch. After heating, the extracts were allowed to cool to room temperature and thoroughly mixed to obtain a uniform solution. The resulting extract was then diluted 10-fold with DI water prior to analysis. For color development, 15 μL of the diluted extract was transferred into a microplate well and mixed with 150 μL of DI water. After initial mixing, 3 μL of 1 N acetic acid and 8 μL of iodine solution (0.2% iodine and 2% potassium iodide) were added sequentially, with brief mixing after each addition. The reaction volume was adjusted to 300 μL by adding 124 μL of DI water. Plates were gently shaken and incubated at room temperature in the dark for 30 min to allow full color development. Absorbance was recorded at 620 nm using a Synergy HTX multimode microplate reader (BioTek, Los Angeles, CA, USA). Amylose content was quantified using a calibration curve generated from standards ranging from 0 to 35%.

Gelatinization properties were evaluated using a differential scanning calorimeter (DSC model 4000, PerkinElmer, Boston, MA, USA) according to previously reported procedures. Briefly, 8 mg of rice flour was weighed into stainless-steel DSC pans, and 16 μL of DI water were added. The pans were hermetically sealed and equilibrated at room temperature for 1 h prior to analysis. Samples were then heated from 25 to 125 °C at a scanning rate of 5 °C/min. Gelatinization parameters, including onset (*T*_o_), peak (*T*_p_), end (*T*_e_) temperatures and ΔH were determined using Pyris data analysis software (PerkinElmer Inc., Boston, MA, USA; version 11.1).

### 2.3 Extraction of Volatiles by HS-SPME

#### 2.3.1 Sample Grinding Optimization

To prevent loss of volatiles, different grinding methods were compared (cyclone mill vs Geno/Grinder, with/without liquid nitrogen). The best results came from using the Geno/Grinder (1600 mini G Spex SamplePrep) with liquid nitrogen (LN_2_). Sixty paddy rice seeds were placed in 15 mL polycarbonate vials with one 9.5 mm stainless steel ball per 15 seeds, capped, and precooled in LN_2_ for 6 min. Grinding was performed in a precooled cryo-block at 1500 strokes/min for 1 min followed by 30 s pauses, repeated 6 times, with LN_2_ immersion every 2 cycles to maintain low temperature. This procedure resulted in a homogeneous powder, and the entire ground sample was retained for analysis without any size-based fractionation or selective separation. In contrast, cyclone milling resulted in elevated levels of oxidation-derived compounds, likely because of heat generation during grinding. The cryogenic Geno/Grinder method minimized such artifacts, preserved volatile integrity, and enabled simultaneous processing of up to 15 samples without cross-contamination, thereby improving both analytical reliability and throughput.

#### 2.3.2 HS-SPME GC-MS/MS Quantitative Volatile Analysis

Targeted quantitative GC-MS/MS analysis was performed to characterize volatile profiles of paddy rice samples in duplicate, following the method described by Sen, Hope, Bell, and Lafontaine^26^ with minor optimization. Analyses were conducted using a Shimadzu Nexis GC-2030 (Shimadzu, Japan) system equipped with a GCMS-TQ8050NX triple-quadruple mass selective detector and AOC-6000 plus autosampler (Shimadzu, Japan). Volatiles were separated on a 30 m × 0.25 mm × 0.25 μm HP-5MS UI Agilent J&W GC column. To optimize volatile absorption on the fiber various incubation temperatures, durations, and fiber exposure times were tested. Optimal conditions were identified when rice samples were incubated at 80 °C for 15 min with continuous agitation at 250 rpm, followed by solid phase micro extraction for 15 min using a divinylbenzene/carboxen/polydimethylsiloxane (DVB/CAR/PDMS) SPME fiber at 80 °C. Volatiles were then desorbed in the inlet at 240 °C for 3 min. Helium was used as the carrier gas at a flow rate of 1 mL/min, with a constant inlet pressure of 46.7 kPa. The GC oven temperature program was as follows: 35 °C (5 min), ramped to 100 °C at 5 °C min^–1^, then to 150 °C at 3 °C min^–1^, followed by 160 °C at 8 °C min^–1^, and finally to 250 °C at 25 °C min^–1^, with a final hold of 5 min, resulting in a total run time of 39.5 min.

Targeted quantitative analysis of rice volatiles was performed using GC-MS/MS system operated in multiple reaction monitoring (MRM) mode. The method was adapted from a previously validated method,^26^ and further optimized for comprehensive rice aroma profiling. To expand compound coverage, published studies reporting volatile constituents of aromatic and nonaromatic rice were systematically reviewed (Table S1), and a rice-specific target list was assembled. From this data set, several key volatile compounds, either frequently reported or identified as aroma biomarkers, were selected and incorporated into the laboratory’s existing 140 compound MRM method (Table S2).

For these additional compounds, MRM transitions were optimized using product ion scan data and integrated into the acquisition method. The finalized method enabled absolute quantification of approximately 164 volatile compounds across diverse chemical classes, including esters, alcohols, aldehydes, terpenes, thiols, carboxylic acids, and pyrazines (Table S2). Quantification of each compound was achieved using external calibration with internal standard correction. Calibration curves were generated by injecting 10 μL of ten concentration levels (approximately 1 to 1000 pg/ul) prepared in ethanol from mixed analytical standards containing all 164 target compounds (Table S2). Linear regression models were constructed for each compound and used for quantitative analysis. To quantify the compounds, 500 ppb of an isotope-labeled internal standard (ISTD) mix (hexanal-d_12_, ethyl hexanoate-d_11_, n-Octyl-d_17_ Alcohol, 2-acetyl-1-pyrroline-d_5_, phenylacetaldehyde-d_5_, linaloold_5_, and β-myrcene-d_6_ Table S2) representing most of the compound classes under study were injected with each calibration point. These internal standards were selected to minimize interference from endogenous compounds, reduce coelution effects, and ensure accurate and reproducible quantification across chemical classes. Compound-specific MRM transitions, calibration equations, coefficients of determination (*R* ^2^), limits of detection (LOD), and limits of quantification (LOQ) are summarized in Table S2. Across the evaluated concentration ranges, the majority of analytes demonstrated strong linear responses, with *R* ^2^ values exceeding 0.97.

For sample analysis, approximately 500 ± 0.1 mg of finely ground paddy rice was weighed into 10 mL headspace vials, and 5 μL of the ISTD mixture (1 ng μL^–1^) was added prior to analysis. Method performance was monitored by analyzing a midpoint calibration standard and an internal standard-only blank at both the beginning and end of each analytical batch (five samples analyzed in duplicate). Measured concentrations were compared against expected values, and relative standard deviation (RSD) values below 20% were considered acceptable. If this criterion was not met, fresh calibration standards and internal standards were prepared, and the affected samples were reanalyzed. Quantification of target volatiles in rice samples was performed using compound-specific calibration curves and the most appropriate internal standard assigned based on chemical class. Final concentrations are reported as mg kg^–1^ of rice.

### 2.4 Sensory Evaluation

Prior to evaluation, panelists were familiarized with 15 aroma reference standards, which were selected based on a review of published literature on rice aroma chemistry and sensory. The trained panel of 10–15 participants completed the sensory study at the Food Science Department of the University of Arkansas over ∼16 days. Nine samples were evaluated per session, including a consistent control sample (Aroma 22) to provide an internal reference across evaluation days. For sample preparation, 1 g of ground paddy rice was weighed into a glass vial, and 1.3 mL of DI water was added to hydrate the grain powder. This hydration step was optimized during method development, as the absence of water resulted in poor sensory discrimination among samples. The addition of water facilitated more uniform heat transfer and partial matrix softening, which enhanced the release of volatile compounds into the headspace and improved aroma detectability. The selected 1:1.3 (w/v) rice-to-water ratio provided the best balance between volatile release and sensory contrast among genotypes, whereas higher hydration levels led to reduced differentiation, likely due to dilution-driven changes in headspace partitioning. This ratio is also consistent with commonly reported conditions in rice cooking and sensory studies.^27–30^ Samples were heated in a sand bath at 80 °C for 30 min (Figure S1), after which they were evaluated immediately. Panelists independently smelled each sample and recorded their responses on the provided sensory sheet (Figure S2). Panelists ranked samples according to aroma intensity, from least to most intense, by placing the corresponding sample number along a predefined intensity scale. Aroma quality was further characterized using a Check All That Apply (CATA) approach, in which panelists selected all applicable descriptors from the predefined list of 15 aroma terms that best matched the perceived aroma profile and recorded the sample name in the designated space. Responses were recorded on paper and then entered into a Qualtrics survey (Provo, UT, USA) on Samsung Galaxy Tab A7 Lite Android tablets (Suwon, South Korea) for data capture and analysis. Ethical permission was evaluated, and the study protocol (IRB project no. 2303461820) was granted exemption by the University of Arkansas Institutional Review Board. All panelists provided informed consent prior to participation.

### 2.5 Statistical Analysis

Aroma intensity differences among rice genotypes were evaluated by using least significant difference (LSD) comparisons. Aroma intensity values were bias-adjusted using linear mixed-effect models implemented in R (version 4.3.2) via RStudio to account for experimental variability inherent to sensory evaluation. The day of evaluation and panelist were included as random effects to correct for day-to-day fluctuations and individual assessor bias, ensuring that observed differences in aroma intensity primarily reflected genotype-dependent effects rather than sensory or environmental noise. Genotype-specific effects were estimated as best linear unbiased predictors (BLUPs) and combined with estimated marginal means to obtain bias-adjusted aroma intensity values for a robust comparison among rice genotypes.

Following bias adjustment of aroma intensity, CATA (Check-All-That-Apply) data were summarized to characterize aroma quality at the descriptor level. For each evaluation day and rice sample, CATA responses were aggregated by computing citation frequency for each descriptor. Citation frequency for each day x sample x descriptor was calculated as the number of panelists selecting a descriptor divided by the total number of panelists who evaluated that sample, yielding a proportional measure of descriptor occurrence. Within each evaluation day, descriptor-level differences among genotypes were assessed using Cochran’s Q test. Descriptors that did not show significant genotype-dependent differences on any evaluation day (*p* > 0.1) were excluded from subsequent sensory and multivariate analyses.

To visualize patterns in aroma intensity and sensory descriptors across genotypes, a heatmap was generated in R using the pheatmap package. The control sample (R127, ARoma 22), which was evaluated across multiple days, was summarized as a single reference profile by averaging its numeric sensory variables across days prior to visualization. The resulting data matrix (samples × sensory variables) was column-scaled (z-score) before clustering, and hierarchical clustering was performed using Euclidean distance and Ward’s method (ward.D2) for both rows and columns. To link sensory outcomes with chemical composition, correlation analysis was conducted between sensory variables (aroma intensity and descriptor frequencies) and volatile compounds quantified using GC-MS. Correlations with *p* < 0.05 were considered statistically significant. For metabolomics-based feature assessment, one-way ANOVA was applied to targeted volatile compound abundance values to identify compounds differing significantly among genotypes (*p < 0.05*); all quantified compounds met this criterion. Hierarchical clustering was additionally performed on the metabolomics data to identify volatile compounds associated with aroma intensity and sensory descriptors. Finally, to integrate sensory and chemical data sets and visualize sample relationships across multiple data blocks, Multiple Factor Analysis (MFA) was performed in R (v4.3.2) using the FactoMineR package.^31^

## 3 Results and Discussion

### 3.1 Sensory Aroma Intensity Variation across Rice Genotype

Sensory aroma intensity was evaluated using a continuous linear scale (0 = no detectable aroma; 100 = extremely intense) (Table S4), enabling quantitative differentiation among genotypes with substantially higher resolution than binary classification approaches such as the traditional 1.7% KOH test.^23^ This scale was selected because the study aimed to quantify aroma intensity rather than consumer preference, continuous scales provide greater sensitivity for detecting subtle differences among samples and facilitate integration with quantitative volatile data. A reference control cultivar, ARoma 22 (R127), was included in every evaluation session to serve as an internal benchmark and to monitor panel consistency across days. The inclusion of this control enabled bias adjustment and improved the reliability of comparisons across the 16-day evaluation period.

Across the evaluated panel (*n* = 126), substantial phenotypic diversity in perceived aroma expression was observed, with unadjusted aroma intensity values ranging from 24.2 to 67.0, representing an approximately 2.8-fold difference between the lowest and highest aroma intensity genotypes (Table S4; Figure 1). This wide phenotypic range indicates that the sensory method was sufficiently sensitive to discriminate among genotypes spanning weak to very strong aroma expression, supporting its utility for both forward selection of high-aroma lines and counter-selection against low-aroma backgrounds in breeding populations. Least-squares mean (LSMean) separation of unadjusted aroma intensity revealed clear and statistically significant differences among genotypes (*p* < 0.05; Table S4). ARoma 22 (R127) exhibited high-aroma intensity (LSMean = 61.7) and grouped within the highest LSD class (“A”), confirming its suitability as a high-aroma intensity control. Twenty-four genotypes were assigned to this top intensity class, while an additional 18 genotypes formed the next highest class (“B”), reflecting significantly lower but still elevated aroma intensity relative to the top group (*p* < 0.05). The remaining genotypes were distributed across progressively lower LSD classes corresponding to weak/moderate aroma expressions. Overall, approximately 33% of the evaluated genotypes fell within the top two aroma intensity classes, indicating that substantial genetic diversity for strong aroma expression exists beyond the limited set of commercially available aromatic cultivars.

**Figure 1:**
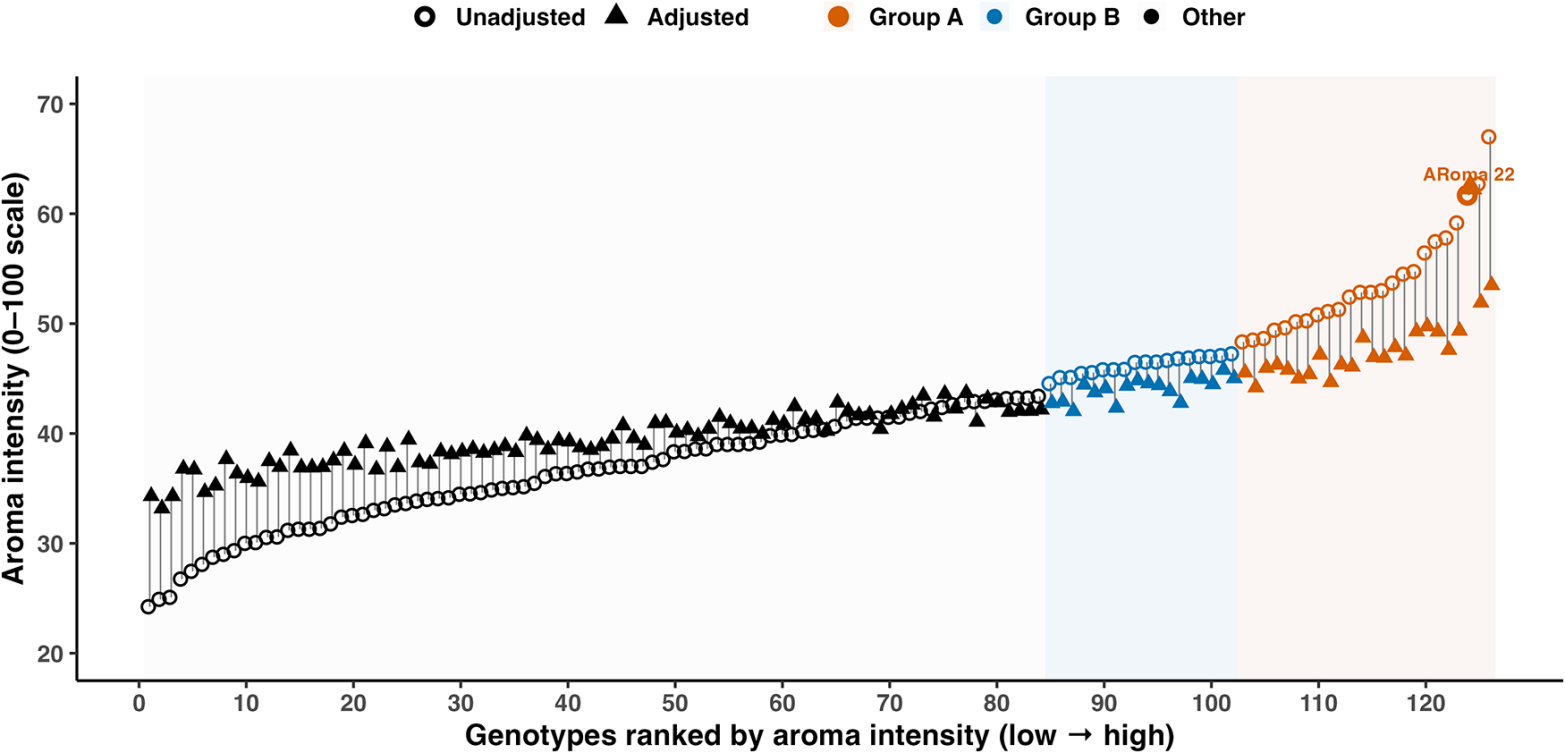
Comparison of unadjusted and bias-adjusted aroma intensity across rice genotypes.

To reduce experimental noise inherent to sensory testing, aroma intensity values were bias-adjusted by using a linear mixed-effects model with day and panelist included as random effects. Bias-adjusted aroma intensity values ranged from 33.2 to 62.4 (Figure 1), representing a modest compression of the range relative to unadjusted values. Importantly, bias adjustment had minimal impact on genotype ranking as unadjusted and bias-adjusted aroma intensity values were strongly correlated (Pearson *r* = 0.96, *p* < 0.001). This result indicates that bias adjustment primarily improved measurement precision rather than altering conclusions regarding genotype performance while also validating the internal consistency of the sensory panel. As bias-adjusted values provide more accurate effect-size estimates for breeding value prediction, they were used for all subsequent multivariate and correlation analyses.

### 3.2 Volatile Composition and Class-Level Distribution in Paddy Rice

The volatile profiles of the 126 rice cultivars were characterized using targeted SPME-GC-MS/MS analysis in MRM mode to assess 164 VOCs in each (Table S2). Of these, 78 compounds were quantified in one or more cultivars, whereas the remaining compounds were not detected in at least one cultivar, indicating that they were either absent or present at concentrations below the analytical detection limits. The quantified VOCs comprised 19 alcohols, 18 aldehydes, 15 esters, 7 terpenes, 5 ketones, 5 hydrocarbons, 3 pyrazines, 3 nitrogen-containing heterocyclic compounds, and 1 compound each of furan, phenolic, and organic acid classes. To evaluate the relative contribution of each chemical class to the overall volatile profile, class-level distributions were assessed by summing the absolute concentrations of individual compounds within each chemical class and normalizing these totals to the sum of all quantified volatiles per genotype. This approach provides insight into which compound classes dominate the volatile pool on a mass basis, although it should be noted that this does not directly reflect sensory impact, as many aroma-active compounds exert strong sensory effects at trace concentrations due to their exceptionally low odor thresholds.

Across the studied genotype panel, alcohols represented the dominant class, accounting for an average of 59.6% of the total volatiles, followed by aldehydes (21.0%), hydrocarbons (8.3%), esters (3.4%), ketones (3.3%), and furans (1.9%). Minor classes collectively contributed less than 2.5% of the volatile pool and included terpenes (1.2%), organic acids (0.56%), nitrogen-containing heterocycles (0.3%), phenolics (0.15%), and pyrazines (0.03%) (Figure 2). Alcohols, aldehydes, and hydrocarbons have consistently been reported as major volatile classes in rice; however, their relative abundances vary across studies. For example, hydrocarbons were reported as the predominant class in raw milled rice by Zhao et al.,^32^ whereas Xiong et al.^33^ identified aldehydes and alcohols as the most prevalent classes, with ketones also contributing substantially to the volatile profile. The differences in dominant volatile classes among studies likely reflect variations in the sample matrix. In this study, the use of intact paddy rice is likely to account for the high relative abundance of alcohols compared to aldehydes. In particular, the use of intact paddy rice in the present study, rather than milled rice, may favor a higher relative alcohol abundance due to critical differences in lipid oxidation dynamics between intact and milled rice.^34^

**Figure 2:**
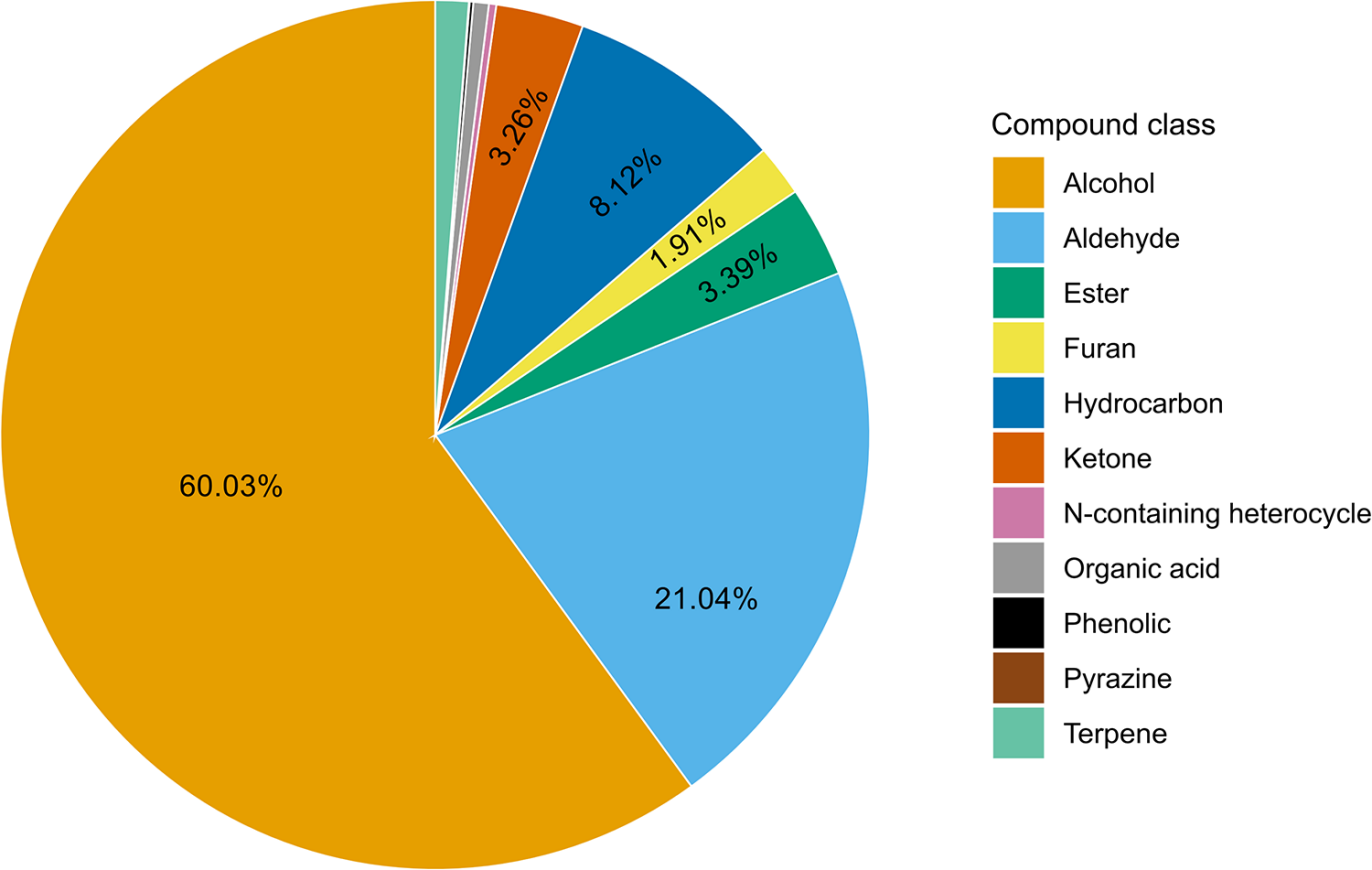
Relative contribution of each volatile compound classes to the overall aroma profile of 126 rice samples.

### 3.3 Correlations between Aroma Intensity, Sensory Descriptors, and Volatile Compounds

To link chemical data with sensory perception, correlations among aroma intensity, sensory descriptors, and individual VOCs were examined (Table S5). Prior to correlation analysis, sensory descriptors were evaluated for genotype-dependent variation using Cochran’s Q test. Among the 15 aroma descriptors initially evaluated, citrus and herbal did not exhibit significant genotype effects at any evaluation day (*p* > 0.1) (Table S6) and were therefore excluded from further analysis.

Correlation analysis revealed that a statistically significant positive correlation existed between overall aroma intensity and the popcorn descriptor (*r* = 0.43, *p* < 0.001), indicating that panelists perceived higher overall aroma intensities in samples exhibiting increased popcorn-like character. Among all volatiles, 2AP exhibited the highest and statistically significant positive correlation with both overall aroma intensity (*r* = 0.5, *p* < 0.001) and the popcorn descriptor (*r* = 0.41, *p* < 0.001), confirming its central role in shaping classical aromatic rice sensory perception.

These findings are consistent with previous reports in which sensory scores derived from chewing and inhalation were positively correlated with 2AP concentration.^33^ Despite its relatively low abundance, the strong sensory impact of 2AP observed here reflects its exceptionally low odor threshold (≈0.1 ppb in water) and highly distinctive popcorn-like aroma,^35,36^ further validating the sensitivity of the sensory phenotyping approach employed in this study. However, correlation analysis indicated that 2AP was not the sole contributor to elevated aroma intensity.

Several additional volatiles also showed significant positive correlations (although less than 2-AP), including nonanal (*r* = 0.29), isobutyl alcohol (*r* = 0.26), 1-heptanol (*r* = 0.25), 2-pentanone (*r* = 0.23), ethyl hexanoate (*r* = 0.22), phenylacetaldehyde (*r* = 0.22), ethyl octanoate (*r* = 0.21), phenylethyl alcohol (*r* = 0.19), 1-pentanol (*r* = 0.19), and indole (*r* = 0.19). Collectively, these compounds span multiple chemical classes and are associated with fruity, floral, alcoholic, and fermented sensory notes, suggesting that an elevated aroma intensity arises from the combined contribution of multiple odor-active compounds rather than from 2AP alone. Among these, pentan-1-ol has previously been reported to correlate positively with sensory scores^33^ and has been suggested to contribute popcorn-like notes in nonfragrant rice varieties lacking 2AP.^5^ However, we did not observe a significant correlation between pentan-1-ol and the popcorn descriptor, suggesting that while 1-pentanol may contribute to overall aroma intensity rather than popcorn specifically.

In addition, several volatiles exhibited significant negative correlations with aroma intensity, including acetophenone (*r* = −0.239), trans-2-nonenal (*r* = −0.214), α-terpineol (*r* = −0.216), dihydrocarveol (*r* = −0.202), and menthol (*r* = −0.199) (Table S5). These compounds are commonly associated with woody, rancid, or fatty notes and may suppress or mask desirable aromatic perception when present at higher levels. Together, these results indicate that while 2AP is a major driver of aroma intensity, the overall sensory expression of aromatic rice reflects a balance between positively contributing volatiles and compounds that negatively influence perceived aroma strength. This suggests that achieving superior aroma quality requires not only selecting for high levels of desirable compounds but also minimizing or eliminating the accumulation of negative aroma contributors.

### 3.4 HCA of Sensory Aroma Profiles and Their Chemical Associations

To identify patterns in the complex sensory and chemical data and classify genotypes on the basis of their integrated aroma profiles, HCA was performed. HCA classified the 126 evaluated genotypes into five distinct sensory-chemical groups (clusters I–V) based on their perceived aroma characteristics (Figure 3). Each cluster exhibited characteristic sensory attributes and chemical signatures that distinguished it from the other groups.

**Figure 3:**
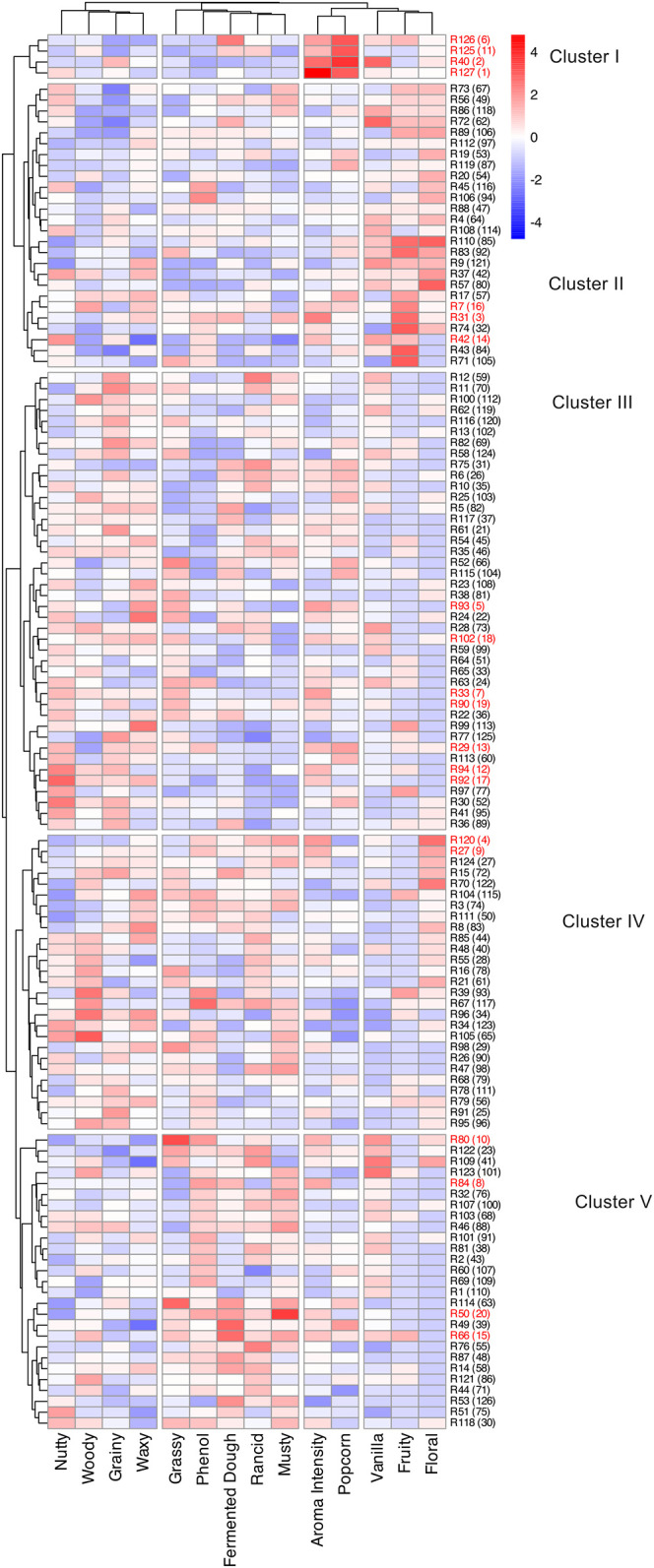
Hierarchical cluster analysis (HCA) of rice genotypes based on sensory aroma attributes. Data were auto scaled by attribute prior to clustering. Rows represent genotypes, with ranks shown in parentheses based on bias-adjusted aroma intensity, and columns represent sensory aroma attributes. Color intensity reflects relative sensory perception, with red indicating higher than average intensity and blue indicating lower than average intensity. Genotypes labeled in red font denote the top 20 genotypes with the highest overall aroma intensity.

#### 3.4.1 Cluster I: Popcorn-Dominant Genotypes

Cluster I was uniquely characterized by significantly higher popcorn aroma intensity and highest overall aroma intensity compared with all other clusters (Tukey HSD, *p* < 0.001). This cluster comprised four genotypes (R40, R125, R126, and R127 (the reference control ARoma 22)), all of which ranked in the top 20 for overall aroma intensity. These genotypes shared a strong popcorn-like aroma signature, setting them apart from all other clusters.

Within cluster I, genotypes R127 (ARoma 22) and R40 exhibited both high popcorn aroma perception and elevated 2AP concentrations (0.316 and 0.125 ppm, respectively), consistent with the established role of 2AP as the primary molecular driver of popcorn-like sensory perception in aromatic rice. Notably, 2-AP is predominantly localized in the outer grain tissues, particularly the bran and aleurone layer, suggesting that the use of whole paddy rice in the present study enables effective capture of this key aroma contributor.^37^ In addition to its popcorn character, genotype R40 exhibited a pronounced vanilla-like note in its aroma profile, suggesting the presence of vanillin or structurally related sweet phenolic compounds such as vanillin, ethyl vanillin, or methyl vanillin. These compounds have been reported in aromatic rice cultivars and are known to contribute sweet, dessert-like nuances that complement the popcorn character.^38,39^ The co-occurrence of popcorn and vanilla notes in R40 may enhance consumer appeal, warranting further evaluation through consumer testing. Correlation analysis revealed that 2-AP was positively associated with several other volatiles, including nonanol (*r* = 0.39), 1-heptanol (*r* = 0.20), isobutyl alcohol (*r* = 0.20), and 2,2,4,6,6-pentamethylheptane (*r* = 0.26) (*p* < 0.05, Table S5).

Among these, 2,2,4,6,6-pentamethylheptane is particularly important, as it has previously been proposed as a potential volatile biomarker for aromatic rice and shown to covary with 2AP.^40^ The mechanistic basis for this covariation is not fully understood but may reflect shared biosynthetic precursors, coregulation of metabolic pathways, or common environmental/genetic factors that influence both 2AP synthesis (via the betaine aldehyde dehydrogenase 2 (*BADH2*) pathway) and hydrocarbon metabolism.

Interestingly, genotypes R125 and R126 showed similarly strong popcorn perception despite substantially lower 2-AP concentrations (0.0018 and 0.0036 ppm, respectively), which are approximately 100-fold lower than those observed in R127 and R40. This indicates that popcorn-associated aroma perception in these genotypes is not primarily driven by 2AP but rather is likely supported by additional volatile contributors that either mimic the sensory character of 2AP or synergistically enhance popcorn perception.^41^ Several volatile compounds exhibited significant positive correlations with the popcorn descriptor beyond 2AP, including phenylethyl alcohol, ethyl 3-hydroxybutyrate, hexanoic acid, nonanol, 1-heptanol, ethyl octanoate, ethyl benzoate, 2-pentanone, and 1-hexanol (*r* = 0.178–0.262) (Table S5). While these compounds are not individually characterized by popcorn-like odor notes, their combined presence may enhance buttery and roasted grain facets of popcorn aroma through additive or synergistic mixture effects. Sensory descriptors such as woody, musty, and phenolic exhibited negative associations (*r* = −0.279 to −0.383) with popcorn, indicating that these aroma qualities may suppress or mask popcorn notes in rice. This antagonistic relationship suggests that breeding strategies aimed at maximizing popcorn character should not only select for high 2AP or supporting volatiles but also counterselect against genotypes that accumulate woody, musty, or phenolic off-flavors.

In addition to its strong popcorn aroma, genotype R126 was characterized by pronounced fermented dough and fruity notes. The fruity character is consistent with elevated ester content in this genotype (Figure S3), while the fermented dough perception may be associated with higher levels of higher alcohols (e.g., isobutyl alcohol, isoamyl alcohol, phenylethyl alcohol) and aldehydes such as isobutyraldehyde and phenylacetaldehyde. These compounds are widely reported contributors to fermentation-related aroma in rice-based products, including sake and koji^42^ and collectively impart characteristic fermented sensory attributes.^42,43^ The presence of elevated fermentation-associated volatiles in R126, despite it being an unfermented paddy rice sample, suggests that these compounds may arise endogenously through natural enzymatic processes in the grain or during postharvest handling, microbial activity. Overall, cluster I demonstrates that classical popcorn aroma can be achieved through high 2AP levels, through alternative volatile contributors, or through combinations of both mechanisms. The identification of R125 and R126 as strong popcorn genotypes, despite low 2AP, underscores the complexity of aroma perception and the need for breeding programs to consider full volatile profiles rather than relying solely on 2AP as a selection criterion.

#### 3.4.2 Cluster II: Fruity-Floral Genotypes Dominated by Esters and Alcohols

Cluster II was characterized by the highest mean intensities for fruity and floral sensory attributes among all clusters, distinguishing it clearly from the popcorn-dominant cluster I. Three genotypes within this cluster R31, R42, and R7 ranked among the top 20 for overall aroma intensity despite lacking the characteristic popcorn aroma (Figure 3). This finding challenges the current assumption that high-aroma intensity in rice is synonymous with popcorn character and demonstrates that alternative sensory profiles driven by different chemical compositions can achieve an equally strong overall aroma perception.

The fruity-floral sensory profile of cluster II genotypes corresponded with elevated concentrations of volatile esters and alcohols commonly associated with pleasant fruit and floral notes across diverse food systems. Some of these compounds may also originate from outer grain tissues, which have been shown to contain diverse odor-active compounds including lactones and alcohols.^37^ Genotype R31, which ranked third in overall aroma intensity, exhibited exceptionally high levels of short- and medium-chain fatty acid ethyl esters, specifically ethyl hexanoate (0.204 ppm) and ethyl octanoate (0.032 ppm) (Table S5 and Figure 4). These esters are known to possess low odor thresholds^44^ and are known to impart sweet, fruity, pineapple, and banana-like notes.^45^ In addition to esters, R31 showed elevated concentrations of primary alcohols, including 1-hexanol (6.67 ppm) and 1-pentanol (1.605 ppm), alcohols associated with fresh, green, and fruity nuances. These compounds might be acting synergistically with ketones such as 2-pentanone (0.059 ppm) and 2-heptanone (0.155 ppm) to enhance overall fruity perception.^45^ R31 also contained markedly higher levels of 2-pentylfuran (0.349 ppm), a compound previously reported to contribute floral, fruity, nutty, and caramel-like notes in rice (Hinge et al., 2016). 2-Pentylfuran is formed through thermal degradation and oxidation of lipids and has been identified as an important biomarker for the aroma of cooked rice. Additionally, heptanal, which has been associated with sweet, fresh, and penetrating fruity-like aromas in rice, was present at elevated levels in both R31 and R42 (0.039 ppm) compared with the other genotypes evaluated.^45,46^ Interestingly, even genotypes with comparatively lower overall aroma intensity, such as R71, were perceived as strongly fruity, likely driven by high concentrations of specific esters including ethyl 2-butenoate and methyl hexanoate (Figure S3). Similarly, genotypes R72, R73, and R74, which also exhibited strong fruity perception, were characterized by elevated ester abundance relative to other chemical classes (Figure S3).

**Figure 4:**
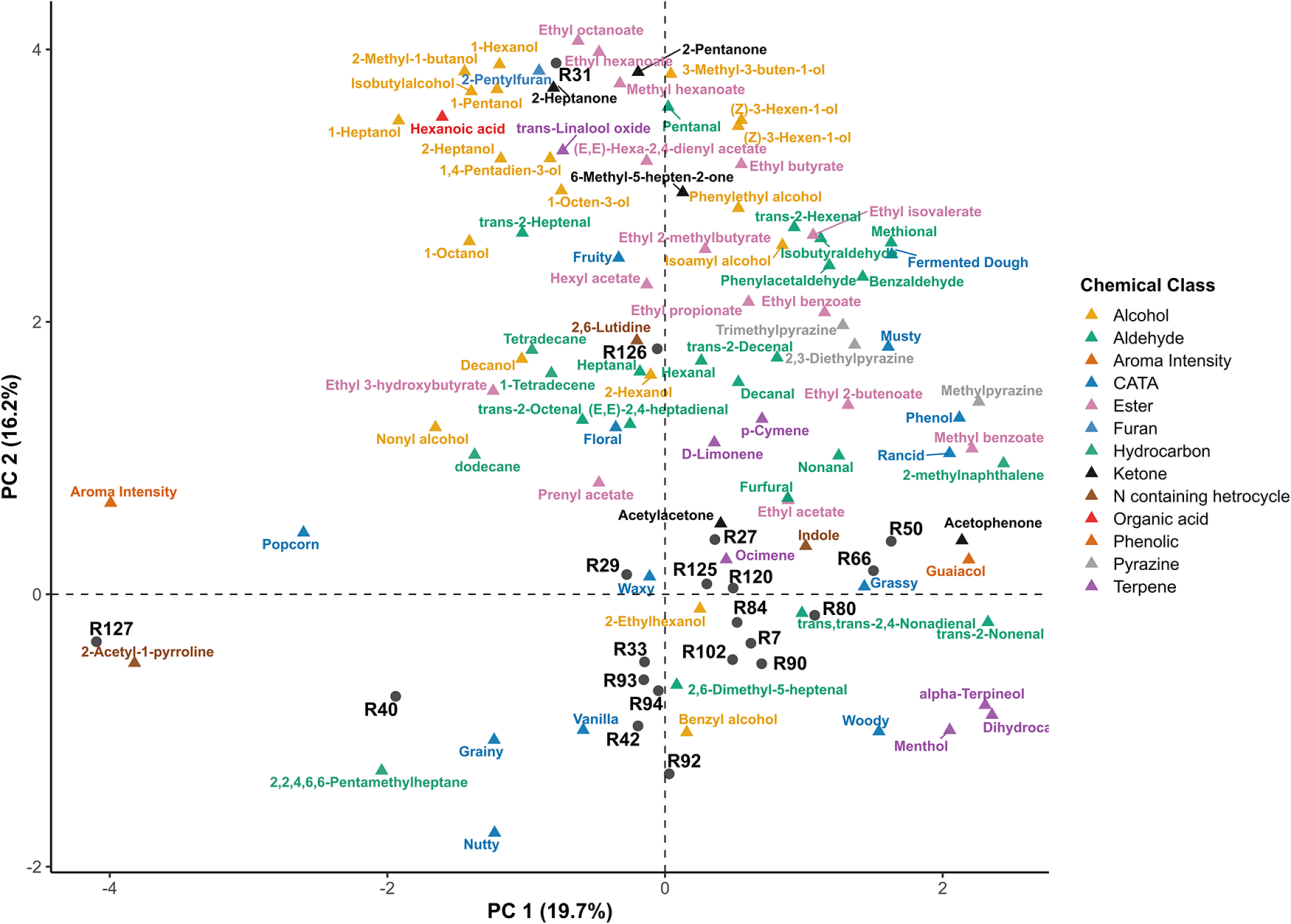
Multiple factor analysis (MFA) of the top 20 rice genotypes with the highest overall aroma intensity, integrating sensory aroma attributes and volatile compound data.

Within cluster II, two genotypes (R110 and R57) exhibited the highest floral intensity among all evaluated genotypes. Floral perception in these genotypes is likely associated with elevated levels of 2-ethylhexanol, a compound known for its sweet, citrus-like, oily, and fresh floral aroma.^47^ Genotype R110 contained the highest concentration of 2-ethylhexanol observed in the entire panel (0.168 ppm), while R57 also contained this compound at levels (0.117 ppm) within its reported odor threshold range (0.075–0.138 ppm).^48^ The presence of 2-ethylhexanol at these concentrations likely contributed substantially to the pronounced floral perception in these genotypes. In raw milled rice, the concentration of 2-ethylhexanol had been reported to be approximately 26–32 ppb,^47^ whereas in the present study concentrations ranged from 17 to 168 ppb across the panel. This broader range and higher maximum concentration suggest that, in addition to endosperm-derived sources, contributions from outer grain tissues (e.g., hull or bran) may influence 2-ethylhexanol abundance, warranting further investigation into its biochemical origin, stability across environments, and potential suitability as a target trait in rice aroma-focused breeding programs.

#### 3.4.3 Cluster III: Nutty-Grainy-Waxy Genotypes with Moderate Popcorn Character

Cluster III comprised seven genotypes (R93, R33, R94, R29, R92, R102, and R90) that exhibited a relatively high overall aroma intensity. Most genotypes within this cluster were characterized by elevated mean intensities of nutty, grainy, and waxy sensory attributes, with some also displaying a moderate popcorn aroma. These attributes did not show strong associations with individual volatile drivers, suggesting that the sensory profiles of cluster III arise from the combined influence of multiple low-to-moderate contributors, rather than from a single dominant aroma compound. Nutty and grainy attributes, together with popcorn aroma, have been reported to correlate positively with consumer acceptance, with no significant differences in their relationships to preference compared with popcorn aroma (Zhou et al., 2023).

In the present study, nutty and grainy attributes exhibited significant negative correlations with rancid and phenolic notes (Table S5), indicating that their sensory expression may be suppressed in genotypes characterized by elevated levels of oxidation- and degradation-associated compounds, particularly aldehydes and phenolic volatiles. Within cluster III, genotype R29 exhibited the highest 2-AP concentration among cluster members (0.101 ppm) and showed the strongest popcorn intensity within this group. This observation indicates that the sensory approach was sufficiently sensitive to resolve relative differences in popcorn perception under a comparable aroma background. However, popcorn intensity in R29 remained lower than that observed in popcorn-dominant genotypes such as R125 and R126 (cluster I), despite R29 having higher 2-AP levels. This reduced popcorn prominence may be attributed to the concurrent expression of nutty, grainy, and waxy sensory attributes in R29, suggesting that popcorn aroma expression is modulated by interactions among co-occurring sensory attributes rather than by 2-AP abundance alone. This phenomenon, known as competitive perceptual masking or mixture suppression, has been well-documented in sensory science^49^ and underscores the complexity of predicting sensory outcomes from chemical composition alone.

Among the top ten genotypes exhibiting the highest popcorn notes, two additional genotypes (R115 and R52) were also assigned to Cluster III. Interestingly, R115 exhibited the highest concentration of 2,3-pentanedione (0.0367 ppm) among all genotypes evaluated (Table S4 and Figure S3). This α-diketone is a well-recognized aroma-active compound known to impart buttery, creamy, nutty, and toasted popcorn-like nuances in various food matrices. To our knowledge, 2,3-pentanedione has not previously been reported as a quantified aroma-active compound in rice, despite its established contribution to buttery and popcorn-like aromas in a wide range of food products at very low odor thresholds (2.4–4.8 ppb).^50^

#### 3.4.4 Cluster IV: Woody-Dominant Genotypes with High-Aroma Outliers Driven by Indole

Cluster IV exhibited the highest mean intensity for woody aroma; however, genotypes characterized by strong woody notes generally did not display a high overall aroma intensity. This cluster nevertheless contained two high-aroma intensity genotypes (R120 and R27), both of which were distinguished by elevated floral notes (Figure 3). Floral aroma has previously been reported to positively influence consumer acceptance of rice.^5^ Interestingly, R120 showed an exceptionally highest abundance of indole (0.203 ppm) approximately four times higher than other studied genotypes in current research and previously reported in the literature.^36,51^ Given its low odor threshold and known contribution to floral and fruity aroma nuances at low concentrations, indole is commonly used as a supplementary index of rice aroma alongside 2-AP.^32,52^ Floral aroma intensity showed a significant positive correlation with indole concentration (*r* = 0.247, *p* < 0.01), supporting the interpretation that elevated indole levels likely underlie the enhanced floral perception observed in R120 within this otherwise woody-dominant cluster. In addition to indole, genotype R120 also contained elevated levels of 2-ethylhexanol (0.112 ppm), a compound previously suggested as a key driver of floral perception in cluster II genotypes. The combined presence of high indole and elevated 2-ethylhexanol may explain the pronounced and distinctive floral character of R120.

#### 3.4.5 Cluster V: Oxidation and Degradation-Dominant Genotypes with Elevated Rancid, Grassy, Musty Phenolic and Fermented Notes

Cluster V had high mean intensities for grassy, rancid, fermented dough, musty, and phenolic notes. Despite containing four genotypes (R80, R84, R50, and R66) that ranked within the top 20 for overall aroma intensity, this cluster is distinguished by poor aroma quality, as the elevated aroma intensity in these genotypes was driven primarily by compounds associated with oxidative degradation, off-flavors, and unpleasant sensory attributes.

The chemical basis for the undesirable sensory profile of cluster V genotypes was aldehydes, presence of elevated aldehydes particularly markers of lipid peroxidation products (Figure 4), such as nonanal and (E)-2-nonenal, 2,4-nonadienal, (E,E). These compounds derived from fatty acids are formed through different pathways.^53^ The sensory impact of these aldehydes is exacerbated by their low odor thresholds and their tendency to dominate sensory perception even when present alongside more desirable aroma compounds. The use of whole paddy rice in this study likely contributed to the increased detection of these compounds, as outer grain tissues (bran and germ) are enriched in lipids and are therefore more prone to the formation of oxidation-derived volatiles. Correlation analysis revealed that aliphatic aldehydes exhibited strong and highly significant positive intercorrelations, forming a tightly covarying chemical network, likely amplifying the overall rancid and fatty sensory impact. Specifically, hexanal showed robust associations with trans-2-haptenal (*r* = 0.771, *p* < 0.001), trans-2-hexenal (*r* = 0.713, *p* < 0.001), nonanal (*r* = 0.601, *p* < 0.001), and heptanal (*r* = 0.511, *p* < 0.001), while decanal was strongly correlated to heptanal (*r* = 0.620, *p* < 0.001) (Table S5). These findings are in close agreement with Mathure, Jawali, Thengane, and Nadaf,^36^ who reported that various aldehydes form a tightly correlated group in rice cultivars and are indicative of oxidative degradation processes. Importantly, the rancid attribute showed significant positive correlations with dodecane, musty, and fermented dough notes while exhibiting negative correlations with aroma attributes regarded in the literature as desirable, such as fruity, grainy, and nutty (Table S5). The musty attribute seems to be associated with higher relative abundances of alkyl-substituted pyrazines, including trimethylpyrazine, 2,3-diethylpyrazine, and methylpyrazine, supporting the interpretation that combination or higher levels of these pyrazines in rice can produce an undesirable stale, earthy-musty flavor. While often used for their desirable roasted nutty, coffee, and cocoa aromas in food, alkylpyrazines are also recognized as earthy-musty, moldy aroma.^54^ In contrast, the phenolic attribute is likely associated with genotypes exhibiting elevated levels of guaiacol and acetophenone. Guaiacol has been reported to produce smoky notes in rice,^55^ while acetophenone, although sometimes described as sweet or almond-like, likely contributes to phenolic perception when present in combination with guaiacol and other volatiles. The fermented dough attribute was similarly associated with elevated levels of alcohols and aldehydes, consistent with fermentation- and degradation-related aroma signatures.

Collectively, cluster V genotypes occupied the opposite pole of dimension 1 relative to 2-AP driven (such as R127 and R40), indicating a fundamentally distinct aroma space. The aroma profile of cluster V was shaped not only by elevated levels of oxidation- and degradation-related compounds but also by lower levels of aroma-active compounds (esters, 2AP, alcohols) that typically impart favorable aroma notes, allowing negative attributes to dominate sensory perception. Although these compounds contribute higher overall aroma intensity, they are widely associated with negative aroma quality and have been reported to adversely affect consumer acceptance in rice- and cereal-based products due to their heavy, oxidative, and phenolic character.

Taken together, these results indicate that while 2-AP remains a key contributor to classical popcorn aroma, high-aroma intensity and desirable sensory quality are not solely dependent on 2-AP abundance. Several genotypes exhibited strong fruity, floral, nutty, or grainy aroma profiles in the absence of a pronounced popcorn character, driven by distinct combinations of esters, alcohols, indole, and related volatiles. Conversely, genotypes with high total volatile abundance dominated by oxidation- and degradation-associated compounds showed elevated aroma intensity but poor aroma quality. These findings suggest that future rice breeding efforts should move beyond a strictly 2-AP-centric approach and instead prioritize balanced aroma profiles that enhance positive sensory drivers while minimizing compounds linked to negative perception.

## 4 Conclusions and Practical Implications

The phenotyping approach developed in this study enables the direct evaluation of rice aroma from paddy grain, eliminating the need for milling and cooking while requiring minimal sample quantities. This allows simultaneous assessment of aroma intensity and qualitative attributes, providing a practical alternative to conventional cooked-grain sensory evaluations and binary screening methods. By shifting from binary classification to relative ranking of aroma intensity and descriptor profiles, the framework supports more informed selection decisions in breeding programs, enabling prioritization of lines with desirable sensory characteristics while excluding those dominated by off-aromas.

A key advantage of this approach is its adaptability across breeding programs with different levels of infrastructure. In low-resource settings, it can be applied as a sensory-based, high-throughput screening tool for early generation materials. In contrast, programs with analytical capacity can incorporate targeted volatile profiling to resolve the chemical basis of the aroma variation. In addition, the use of whole paddy rice provides a diagnostic advantage by capturing volatile contributions from the outer grain layers. The observed association between lipid oxidation-derived aldehydes and rancid, phenolic, or musty attributes suggests that this approach may enable early identification of genotypes prone to rapid quality deterioration with implications for storage stability and end-use quality.

Application of this framework across a genetically diverse rice panel demonstrated that aromatic expression is not governed by a single chemical determinant. While 2AP remains a key contributor to classical popcorn aroma, several genotypes exhibited strong and desirable aroma profiles in the absence of elevated 2AP levels, supported instead by distinct combinations of esters, alcohols, indole, α-diketone, and other related volatiles. These findings challenge the long-standing reliance on 2AP as the primary selection target in aromatic rice breeding and highlight alternative biochemical routes to high-quality aroma expression

Despite their various advantages, several limitations should be considered. The sensory evaluation was conducted using a trained panel and a descriptive intensity-based approach, which enables sensitive discrimination among genotypes but does not directly reflect the consumer preference. In addition, aroma was assessed from heated ground paddy rice rather than cooked, milled rice and therefore represents a screening proxy rather than a direct measure of eating quality. Furthermore, because whole paddy rice was used, the resulting volatile profiles reflect combined contributions from husk, bran, and endosperm, which may differ from the aroma profile of edible milled rice. Accordingly, this method should be considered a rapid screening tool, and advanced selections require validation under conventional cooking conditions and consumer-based evaluation. Additionally, future work should focus on genetic mapping of non-2AP aroma drivers, evaluation of their stability across environments and postharvest conditions, and integration with consumer preference studies. Overall, this framework provides a foundation for developing next-generation aromatic rice cultivars with an improved sensory diversity and commercial relevance.

## Supplementary Material





## Notes

Sensory evaluation setup, sensory evaluation sheet, and heatmap with hierarchical cluster analysis of rice genotypes based on volatile profiles (PDF)
Literature summary of rice aroma characterization studies; analytical standard information and calibration parameters; sensory attribute definitions and references; grain physicochemical properties, sensory and volatile composition data set for rice genotypes; volatile-sensory correlation analysis; and Cochran’s Q test results (XLSX)
