# Supplementary material for: Developing Quick Screening Method to Identify Rice Cultivars with Unique Aromatic Features
Source: ACS Omega. 2026 Jun 1;11(23):34502–13. doi: 10.1021/acsomega.6c02582 (PMC13280887; doi:10.1021/acsomega.6c02582)
Supplement: Supplementary file 1 [file ao6c02582_si_001.pdf]

Developing Quick Screening Method to Identify Rice Cultivars with Unique Aromatic Features

Heena Rani[a], Rahul Sen[a], Christian De Guzman[b], Scott Lafontaine[a]*

[a] Department of Food Science, University of Arkansas, 2650 N Young Ave, Fayetteville, AR, USA, 72704

[b]Division of Agriculture, Rice Research and Extension Center, University of Arkansas, Stuttgart, AR, USA, 72160

*Email: scottla@uark.edu

Supplemental data includes two files:

1. **Excel spreadsheet with six tables**

- **Table S1**: Summary of previously published studies on rice aroma characterization, including sample type, analytical methodology, and targeted or untargeted volatile profiling approaches.
- **Table S2**: Detailed information for analytical standards and internal standards used for targeted HS-SPME GC-MS/MS quantification, including compound name, CAS number, purity, retention index, MRM transitions, calibration equations, coefficients of determination (R^2), limits of detection (LOD), and limits of quantification (LOQ).
- **Table S3**: Sensory attribute definitions and reference preparations used for descriptive aroma evaluation of rice samples.
- **Table S4**: Sensory and volatile composition dataset for all evaluated rice genotypes. The table reports cultivar code and cluster assignment (based on Fig. 3), along with sensory outcomes including unadjusted and adjusted aroma intensity metrics and intensities for individual sensory attributes. Targeted GC-MS/MS concentrations (ppm) are provided for each genotype across multiple volatile classes, including alcohols, aldehydes, ketones, esters, furans, pyrazines, terpenes, phenolics, acids, and other nitrogen-containing compounds.
- **Table S5**: Correlation coefficients between key volatile compounds and sensory aroma attributes, with significant correaltions ($p < 0.05$) highlighted in yellow.
- **Table S6**: Results of Cochran's Q test evaluating differences in the frequency of citation of sensory attributes across rice genotypes on each evaluation day.

2. **This PDF documents with three figures**

Figure S1: Sensory setup for sensory evaluation, showing ground paddy rice samples heated in a sand bath to generate headspace volatiles prior to sensory analysis.

Figure S2: Sensory evaluation sheet used by panelists for descriptive aroma analysis of rice samples.

Figure S3: Heatmap and hierarchical cluster analysis (HCA) of rice genotypes based on targeted HS-SPME GC-MS/MS volatile profiles. Data were auto scaled by attribute prior to clustering. Rows represent genotypes, with sensory cluster (based on Fig. 3) shown in parentheses based on bias-adjusted aroma intensity and columns represent sensory aroma attributes.

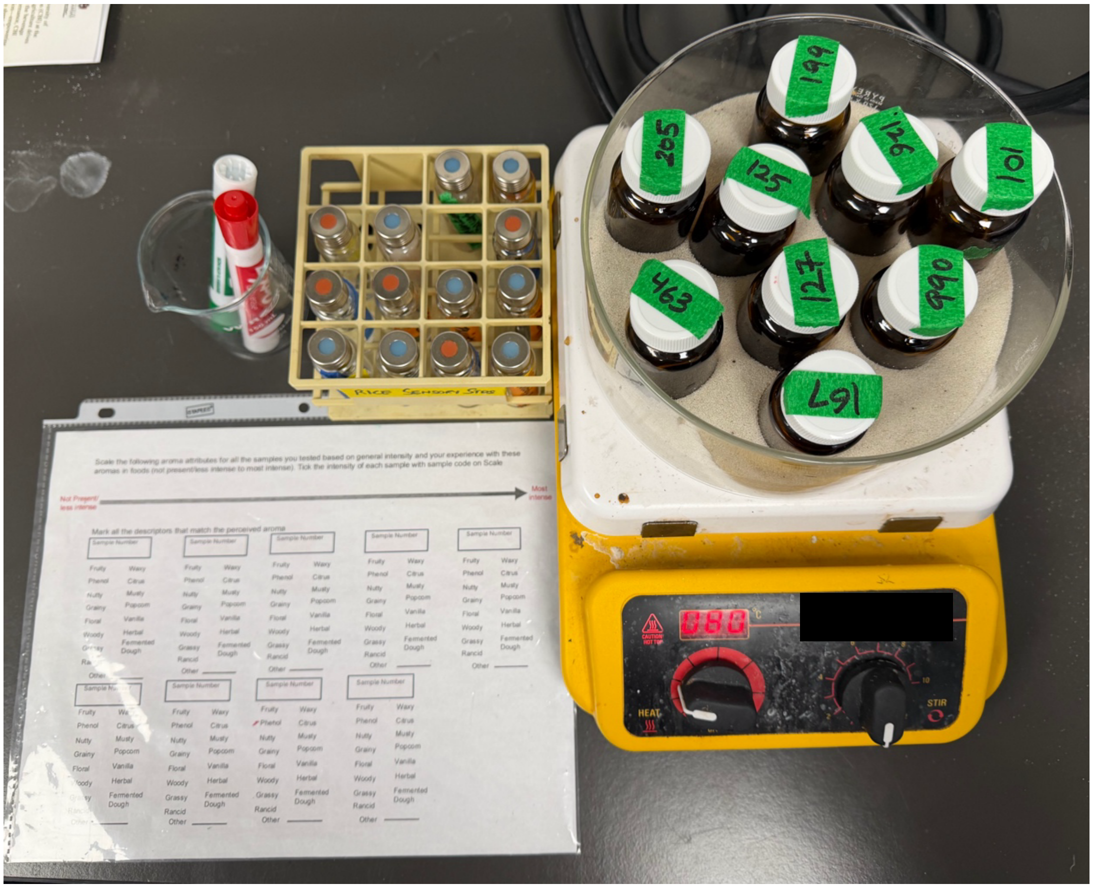

Figure S1: Sensory setup for sensory evaluation, showing ground paddy rice samples heated in a sand bath to generate headspace volatiles prior to sensory analysis.

Scale the following aroma attributes for all the samples you tested based on general intensity and your experience with these aromas in foods (not present/less intense to most intense). Tick the intensity of each sample with sample code on scale

Not Present/ less intense ————————————————————————→ Most intense

Mark all the descriptors that match the perceived aroma

Sample Number	
Fruity	Waxy
Phenol	Citrus
Nutty	Musty
Grainy	Popcorn
Floral	Vanilla
Woody	Herbal
Grassy	Fermented Dough
Rancid	
Other ______	

Sample Number	
Fruity	Waxy
Phenol	Citrus
Nutty	Musty
Grainy	Popcorn
Floral	Vanilla
Woody	Herbal
Grassy	Fermented Dough
Rancid	
Other ______	

Sample Number	
Fruity	Waxy
Phenol	Citrus
Nutty	Musty
Grainy	Popcorn
Floral	Vanilla
Woody	Herbal
Grassy	Fermented Dough
Rancid	
Other ______	

Sample Number	
Fruity	Waxy
Phenol	Citrus
Nutty	Musty
Grainy	Popcorn
Floral	Vanilla
Woody	Herbal
Grassy	Fermented Dough
Rancid	
Other ______	

Sample Number	
Fruity	Waxy
Phenol	Citrus
Nutty	Musty
Grainy	Popcorn
Floral	Vanilla
Woody	Herbal
Grassy	Fermented Dough
Rancid	
Other ______	

Sample Number	
Fruity	Waxy
Phenol	Citrus
Nutty	Musty
Grainy	Popcorn
Floral	Vanilla
Woody	Herbal
Grassy	Fermented Dough
Rancid	
Other ______	

Sample Number	
Fruity	Waxy
Phenol	Citrus
Nutty	Musty
Grainy	Popcorn
Floral	Vanilla
Woody	Herbal
Grassy	Fermented Dough
Rancid	
Other ______	

Sample Number	
Fruity	Waxy
Phenol	Citrus
Nutty	Musty
Grainy	Popcorn
Floral	Vanilla
Woody	Herbal
Grassy	Fermented Dough
Rancid	
Other ______	

Sample Number	
Fruity	Waxy
Phenol	Citrus
Nutty	Musty
Grainy	Popcorn
Floral	Vanilla
Woody	Herbal
Grassy	Fermented Dough
Rancid	
Other ______	

Panelist No ______

Figure S2: Sensory evaluation sheet used by panelists for descriptive aroma analysis of rice samples.

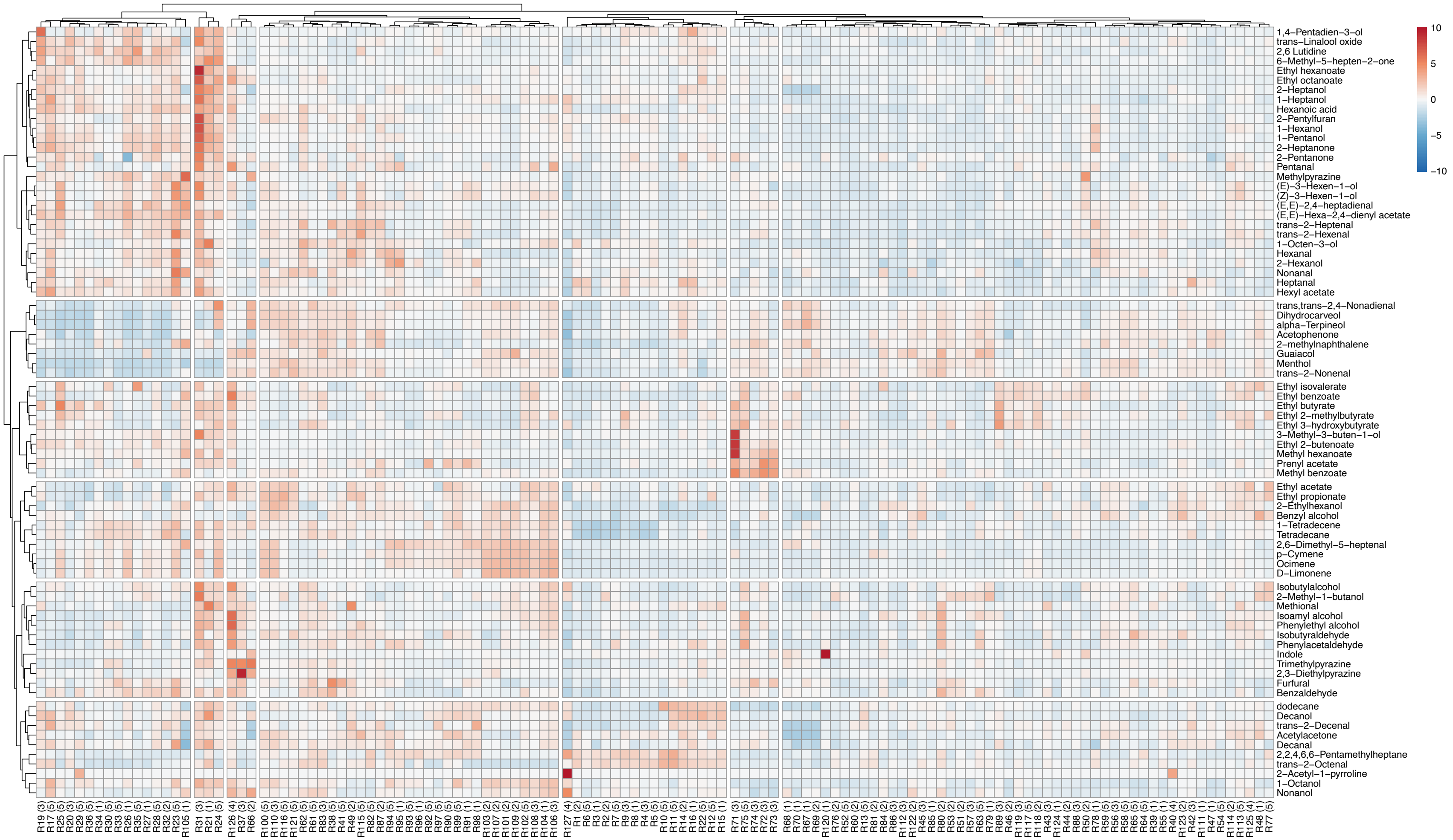

Figure S3: Heatmap and hierarchical cluster analysis (HCA) of rice genotypes based on targeted HS-SPME GC-MS/MS volatile profiles. Data were auto scaled by attribute prior to clustering. Rows represent genotypes, with sensory cluster (based on Fig. 3) shown in parentheses based on bias-adjusted aroma intensity and columns represent sensory aroma attributes.
